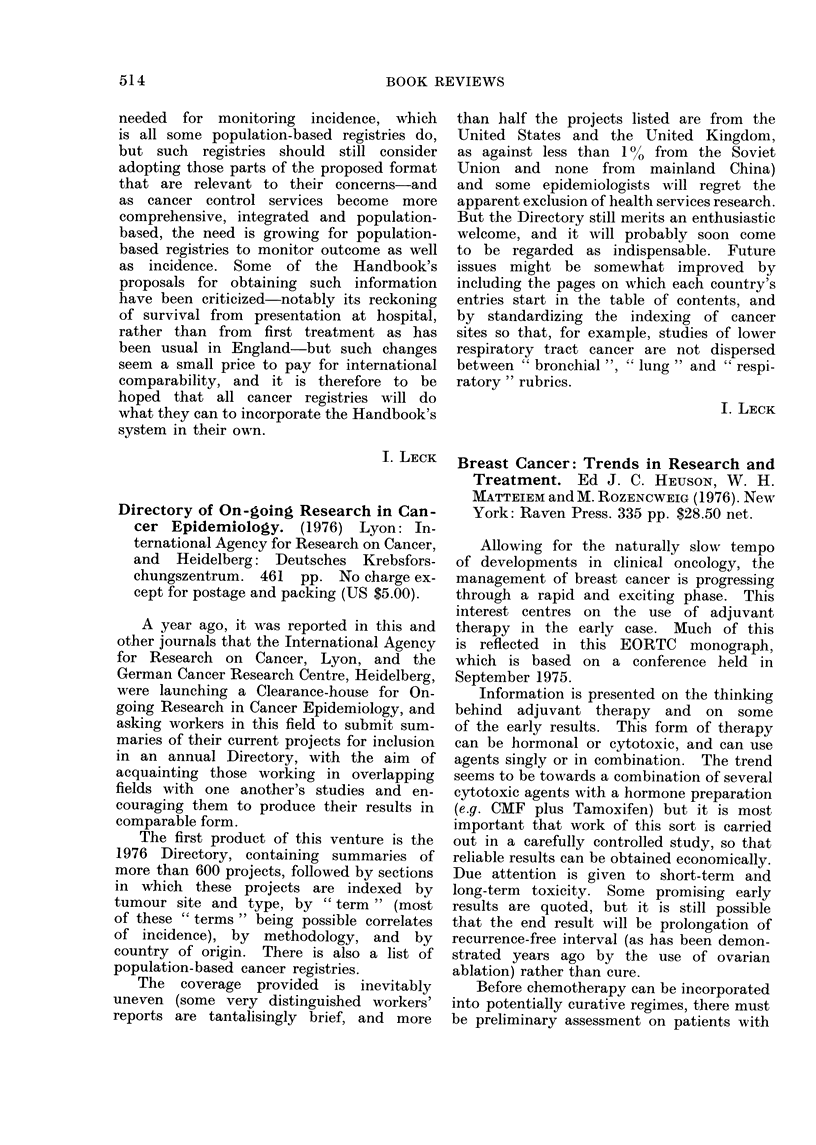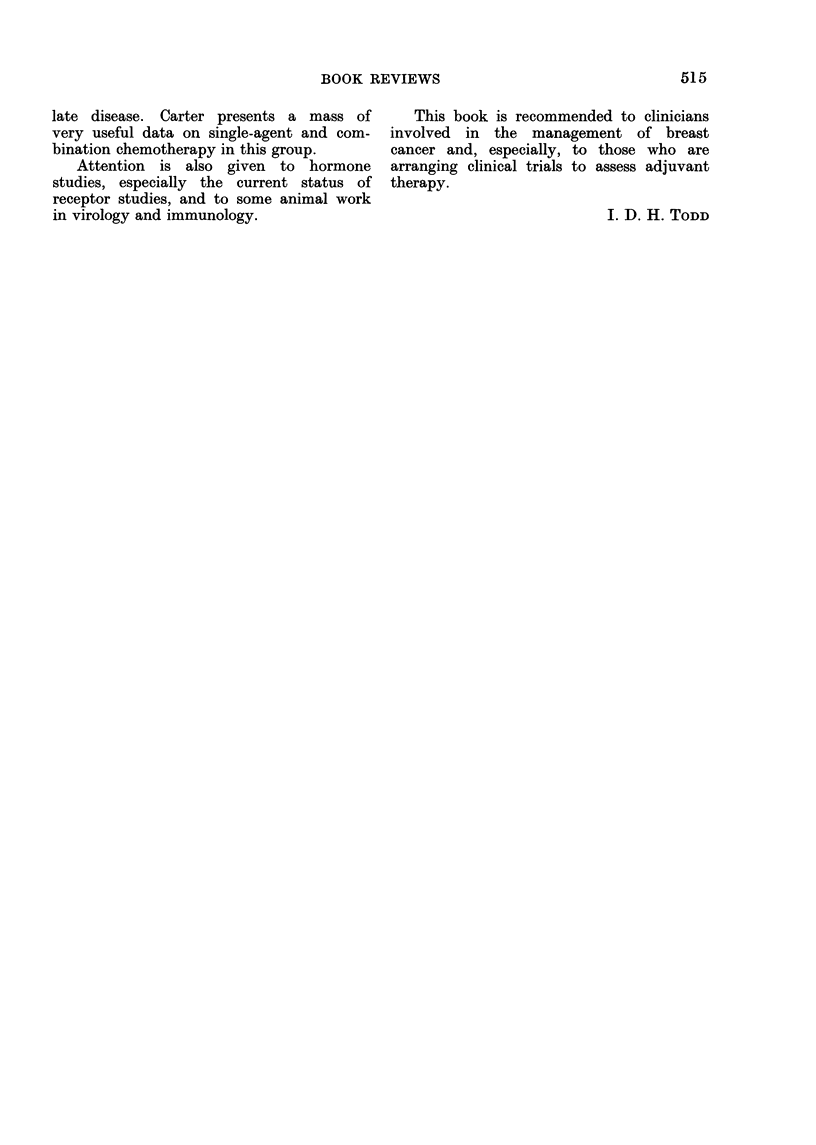# Breast Cancer: Trends in Research and Treatment

**Published:** 1977-04

**Authors:** I. D. H. Todd


**Breast Cancer: Trends in Research and Treatment.** Ed J. C. HEUSON, W. H. MATTEIEM and M. ROZENCWEIG (1976). New York: Raven Press. 335 pp. $28.50 net.

Allowing for the naturally slow tempo of developments in clinical oncology, the management of breast cancer is progressing through a rapid and exciting phase. This interest centres on the use of adjuvant therapy in the early case. Much of this is reflected in this EORTC monograph, which is based on a conference held in September 1975.

Information is presented on the thinking behind adjuvant therapy and on some of the early results. This form of therapy can be hormonal or cytotoxic, and can use agents singly or in combination. The trend seems to be towards a combination of several cytotoxic agents with a hormone preparation (*e.g.* CMF plus Tamoxifen) but it is most important that work of this sort is carried out in a carefully controlled study, so that reliable results can be obtained economically. Due attention is given to short-term and long-term toxicity. Some promising early results are quoted, but it is still possible that the end result will be prolongation of recurrence-free interval (as has been demonstrated years ago by the use of ovarian ablation) rather than cure.

Before chemotherapy can be incorporated into potentially curative regimes, there must be preliminary assessment on patients with late disease. Carter presents a mass of very useful data on single-agent and combination chemotherapy in this group.

Attention is also given to hormone studies, especially the current status of receptor studies, and to some animal work in virology and immunology.

This book is recommended to clinicians involved in the management of breast cancer and, especially, to those who are arranging clinical trials to assess adjuvant therapy.